# Potential mechanism of the effect of heat stress on milk protein synthesis revealed by integrated metabolomic and proteomic analyses

**DOI:** 10.1186/s40104-025-01338-y

**Published:** 2026-02-10

**Authors:** Jia Zeng, Diming Wang, Huizeng Sun, Hongyun Liu, Feng-Qi Zhao, Jianxin Liu

**Affiliations:** 1 Institute of Dairy Science, College of Animal Sciences, Zhejiang University, Hangzhou, China; 2 Zhejiang Key Laboratory of Dairy Cow Genetic Improvement and Milk Quality Research, Zhejiang, China; 3 Department of Animal & Veterinary Sciences, University of Vermont, Burlington, VT USA

**Keywords:** Dairy cow, Heat stress, Milk protein synthesis, Multi-omics

## Abstract

**Background:**

This study was conducted to investigate the impact of varying degrees of heat stress on milk protein synthesis in dairy cows using comprehensive analyses of metabolomics and proteomics. Eighteen dairy cows were subjected to no heat stress (No-HS), mild heat stress (Mild-HS), and moderate heat stress (Mod-HS). Blood and milk samples were collected to determine the content and composition of amino acids (AA), and milk samples were used for metabolomic and proteomic analyses.

**Results:**

Milk protein yield was significantly lower under Mild-HS and Mod-HS than No-HS (*P* < 0.001). During Mild-HS, no significant difference was found in total AA concentration in both arterial (*P* = 0.545) and venous blood (*P* = 0.057), but arterial AA supply to the mammary gland significantly increased (*P* = 0.045) when compared with No-HS. Under Mod-HS, the supply (*P* < 0.001) and uptake (*P* = 0.001) of total AA in the mammary gland decreased significantly, affecting the availability of precursors necessary for milk protein synthesis. Milk metabolomic analysis revealed that Mod-HS significantly impacted nucleotide metabolism, energy metabolism, and protein synthesis processes including translation, folding, and transport. Milk proteomic analysis showed significant downregulation of ribosomal and heat shock proteins which are crucial for protein synthesis and folding.

**Conclusions:**

These findings suggest that heat stress disrupts AA utilization and energy metabolism in the mammary gland, leading to the reduced efficiency in milk protein synthesis and lowered milk protein yield. This study offers valuable insights into the metabolic and proteomic changes in dairy cows under heat stress, highlighting potential strategies to mitigate the adverse effects of heat stress on dairy production and milk quality.

**Supplementary Information:**

The online version contains supplementary material available at 10.1186/s40104-025-01338-y.

## Introduction

Milk protein is one of the most economically valuable components of milk. Heat stress (HS) compromises the overall health of dairy cows and concurrently impairs daily milk protein yield [1]. The mechanisms by which HS interfere with milk protein synthesis are not currently clear, but may include reduction in the turnover of amino acid (AA), competition for AA supply between milk proteins and structural protein synthesis, and degradation of synthesized casein in the mammary gland (MG) [1, 2].

The synthesis of milk proteins in the MG is intricately dependent on the availability of precursors and the capacity of mammary cells to utilize these precursors [3]. Free AA are the primary precursors for milk protein synthesis, and their supply is supplemented by peptide-bound AA in the blood [2]. The AA supply to the MG depends on the plasma AA concentration and mammary plasma flow (MPF) [3]. In our previous study [4], moderate HS significantly reduced MPF to the MG of dairy cows, resulting in the reduced mammary supply of AA (MPF × arterial concentration), thereby limiting the mammary availability of precursors for milk component synthesis. Milk protein synthesis in the MG of heat-stressed cows is adversely impacted by alterations in AA metabolism, attributed to the reduced AA availability [5, 6]. HS alters AA utilization for protein synthesis and reduces protein translation by decreasing the activity of the mechanistic targets of the rapamycin (mTOR) signaling pathway in mammary epithelial cells (MECs) [7]. Despite these findings, there are still significant gaps in understanding of the precise mechanisms by which HS affects AA metabolism and protein synthesis in the MG. Major gaps remain in understanding how HS-driven changes in AA availability are translated into lower milk-protein output at the cellular-pathway level. It is not clear how many of milk protein precursors are obtained by the MG during HS, and which metabolic processes in mammary synthesis of milk proteins are affected by HS.

We hypothesized that HS affects the MG uptake of milk protein precursors and the ability of the MECs to synthesize milk proteins, resulting in reduced protein yield in milk. The milk metabolome may reflect nutrient uptake and endogenous metabolism in the MG [8], whereas milk proteomics analysis serves as a non-invasive indicator of protein changes within MECs [9]. In this study, we investigated the changes of proteins and metabolites involved in the synthesis of milk proteins during HS and elucidate the specific mechanism of the effects of HS on the synthesis of milk proteins by measuring the supply and utilization of AA in MG and by analyzing milk metabolite and protein changes using metabolomic and proteomic multiplexing. The outcome of our study would provide new insights into the precise regulation of milk protein synthesis and the mechanisms that HS exerts its effect on milk protein synthesis.

## Materials and methods

### Animals and experimental design

The experimental design, dietary formula and nutrient composition have been described previously [10]. Briefly, 18 high-yielding Chinese Holstein cows (milk yield = 41.4 ± 0.47 kg/d, days in milk = 207 ± 4.2 d, parity = 2–3; mean ± standard error) were selected and housed within the same barn in a dairy farm. Each cow went through three treatment periods within two months: no HS with a daily average temperature-humidity index (THI) below 68 (No-HS, referred to as thermoneutral), mild HS (Mild-HS, 68 ≤ THI ≤ 79), or moderate HS (Mod-HS, 79 < THI ≤ 88). The rectal temperature was 38.4, 38.8 and 39.7 °C, and respiratory rate was 43.4, 49.2 and 76.0 bpm for the cows under No-HS, Mild-HS and Mod-HS [10], respectively, confirming that the animals experienced distinct physiological HS conditions [4]. In each period, milk yield was recorded for three consecutive days [4], and 50 mL of milk was collected daily at three time points (06:30, 13:00, and 19:00 h), and milk samples were stored at −80 °C for subsequent metabolomics and proteomics analyses. Blood samples were collected three hours after morning feeding and evening feeding, and centrifuged as described previously [4]. They were then stored at −80 °C for AA determination. Equal aliquots of the morning and evening samples were pooled prior to AA analysis.

### Determination of AA profiles of blood and milk samples

Pooled plasma was pretreated according to the modified method of Mackle et al. [11]. Concentrations of AA in the caudal artery, mammary vein, and milk were determined by an AA automatic analyzer (Hitachi High-Tech Technologies Corporation, Tokyo, Japan). The AA concentrations of hydrolyzed blood samples were corrected for losses during hydrolysis according to the procedure of Delgado-Elorduy [12]. Briefly, 500 μL of blood sample was added with an equal volume of sulfosalicylic acid, mixed and centrifuged at 3,000 × *g*, for 15 min at 4 °C; and the supernatant was passed through a 0.22-μm filtration membrane before determination.

The milk samples were pre-treated as follows for measuring AA concentrations. Initially, 5 mL of 6 mol/L HCl (analytical purity) was added into 1 mL of milk sample and then blown for 15 s with a nitrogen blowing instrument. Subsequently, the mixture was baked in an oven at 110 °C for 24 h. The hydrolyzed solution was transferred to a 50-mL centrifuge tube and diluted with distilled water to a final volume of 50 mL. The solution (1 mL) was then blown with nitrogen until dry. The dried sample was diluted with 2 mL of 0.02 mol/L HCl, filtered through a 0.45-μm filter membrane, and 1–1.5 mL of the filtrate was then collected into the injection bottle.

### Determination and analysis of milk metabolites

To detect metabolites, hydrophilic and hydrophobic compounds in milk were extracted separately as reported previously [13]. Linear Ion Trap (LIT) and triple quadrupole (QQQ) scans were performed on a QTRAP^®^ LC–MS/MS System equipped with an Electrospray Ionization (ESI) Turbo Ion-Spray interface in both positive and negative ion modes. The ESI source parameters included a source temperature of 500 °C and ion spray voltages of 5,500 V (positive) and −4,500 V (negative). The sample extracts were analyzed using an LC–ESI–MS/MS system (UPLC, ExionLC AD; MS, QTRAP^®^ System, SCIEX, Framingham, MA, USA). The analytical conditions were similar to those described by Shi et al. [14]. Unsupervised principal component analysis (PCA) was performed by statistics function prcomp within R (www.r-project.org). The metabolomic data was unit variance scaled before unsupervised PCA. Differential analysis, VIP-PLS-DA modelling and hypergeometric pathway enrichment were carried out with limma v3.50.3, MetaboAnalystR v3.2 and SAM v3.0, respectively, while ggplot2 v3.4.0 was used for visualizations.

Differentially abundant metabolites (DAMs) were identified based on variable importance in projection (VIP) scores greater than 1 and a *P*-value less than 0.05, determined by Student’s *t*-test for two-group comparisons and ANOVA for multiple groups. The VIP values were derived from OPLS-DA results using the MetaboAnalystR package, with data log-transformed and mean-centered. A permutation test (200 permutations) was performed to ensure model validity. Identified metabolites were annotated using Kyoto Encyclopedia of Genes and Genomes (KEGG) Compound database (http://www.kegg.jp/kegg/compound/), and the annotated metabolites were then mapped to KEGG Pathway database (http://www.kegg.jp/kegg/pathway.html). Pathways with significantly regulated metabolites were then fed into MSEA (metabolite sets enrichment analysis), and their significance was determined by hypergeometric test’s *P*-value. Metabolites with VIP ≥ 1 and *P* ≤ 0.5 were considered DAMs for population discrimination [15].

### Milk proteomics measurement and analysis

Milk was processed as previously reported [16]. Milk samples were centrifuged at 10,000 × *g* for 15 min at 4 °C to remove fat. The protein concentration of milk samples was determined using the BCA Protein Assay Kit (P0012, Beyotime, Shanghai, China), with bovine serum albumin used as the standard. Within each of the three experimental periods, the 12 individual milk samples were divided into four groups of three cows each (stratified by days in milk and milk yield), and equal volumes from each cow were combined to generate four independent pools per period (*n* = 4 pools per treatment). This within-period pooling strategy is consistent with label-free milk proteomics guidelines [9].

Protein extraction, trypsin digestion, and LC–MS/MS analysis of milk protein samples were performed as described previously [17, 18] for 4D DIA proteomics (diaPASEF) analysis. Liquid chromatography was performed on a nanoElute UHPLC (Bruker Daltonics, Germany). About 200 ng peptides were separated within 40 min at a flow rate of 0.3 μL/min on a commercially available reverse-phase C18 column with an integrated CaptiveSpray Emitter (25 cm × 75 μm ID, 1.6 μm, Aurora Series with CSI, IonOpticks, Australia). The option of match between runs (MBR) was employed to create a spectral library from data-independent acquisition (DIA) data and reanalyzed using this library. The false discovery rate (FDR) was adjusted to < 1% at both protein and precursor ion levels, and the remaining identifications were used for further quantification analysis. Proteomics analyses were conducted in R v4.2.2 with packages tidyverse v1.3.0, limma v3.50.3, ggplot2 v3.4.0 and clusterProfiler v4.6.0. Differentially expressed proteins (DEPs) were identified as those with log_2_FC > 1.5, FDR < 0.1 and* P* < 0.05. The DIA mass spectrometry data were analyzed using DIA-NN software (v1.8.1) with a library-free approach. The search was conducted using the UniProt UP000009136 bovine database (20230404.fasta), which comprises 37,508 sequences. For pathway annotation of the DEPs, Gene Ontology (GO, http://geneontology.org/) and the KEGG, (https://www.genome.jp/kegg/) were utilized. The visualizations were created using R packages (http://www.r-project.org). The KEGG enrichment bar plots were generated online (https://www.omicstudio.cn/tool/124).

### Calculations and statistical analysis

The ratio of mammary AA uptake to milk AA output (U:O) was calculated based on the amount of AA uptake in the MG and AA output in milk. Mammary plasma flow (MPF) has been reported in our previous study [4]. Mammary uptake and utilization of AA were calculated following the method described for glucose utilization reported by Zeng et al. [4] and briefly described as below:

1. Arterial supply of AA (kg/d) = Arterial AA concentration (kg/L) × MPF (L/d);
2. Mammary venous flow (kg/d) = Venous AA concentration (kg/L) × MPF (L/d);
3. Mammary uptake of AA (kg/d) = Arterio-venous difference of AA (kg/L) × MPF (L/d);
4. Clearance rate of AA (L/h) = MPF (L/h) × Arterio-venous difference of AA (kg/L)/Mammary venous concentration of AA (kg/L);
5. Mammary extraction rate of AA (%) = Arterio-venous difference of AA (kg/L)/Mammary arterial supply of AA (kg/L) × 100%.

All statistical analyses were performed in Python 3.11 using the “statsmodels” package (v0.14). Data were analyzed using a mixed-effects model to account for the repeated measures design and individual cow variation. Statistical model as follows:

1 $${y}_{ij}=\mu +{d}_{ij}{+\tau }_{i}{ + {d}_{ij}{\times \tau }_{i}+\delta }_{j}+{\epsilon }_{ij}$$

where ${y}_{ij}$ is the dependent variable of cow *j* in different HS treatment *i*, $\mu$ is the overall mean, ${{d}_{i}}_{j}$ is the continuous covariate days in milk, ${\tau }_{i}$ is the fixed effect of different HS treatment, ${\delta }_{j}$ is the random effect of individuals, which is assumed to follow normal distribution $N(0,\boldsymbol{ }{\boldsymbol{I}}{\sigma }^{2})$, ${\boldsymbol{I}}$ is the identity matrix, and ${\epsilon }_{ij}$ is the random residuals.

Histograms were generated using GraphPad Prism 8 (San Diego, CA, USA, www.graphpad.com). The KEGG maps were prepared using Adobe Illustrator (Adobe Inc., San Jose, CA, USA). Data were expressed as the mean ± standard error of the mean (SEM). The mechanistic diagram was created using BioRender (https://app.biorender.com) to visually illustrate the proposed pathways.

## Results

### Milk yield and AA utilization by MG

**Fig. 1:**
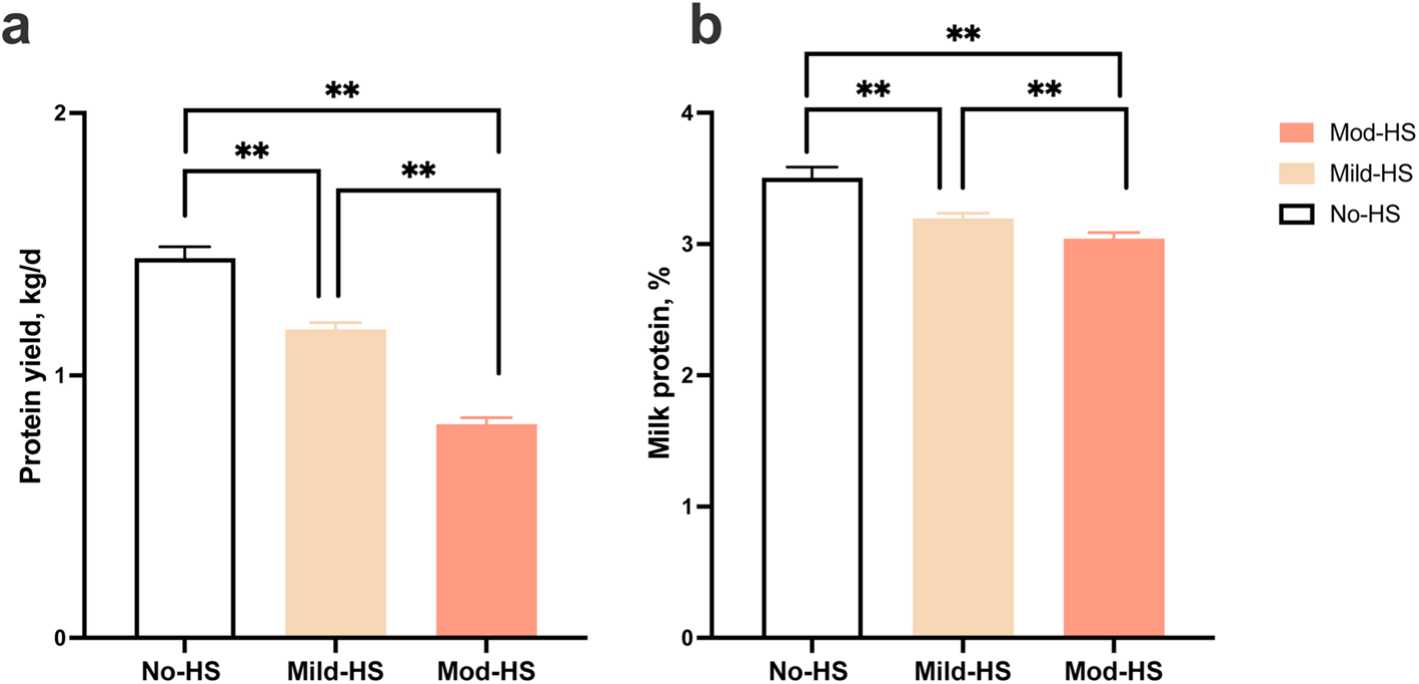
The yield of protein in milk (**a**) and milk protein concentration (**b**) of dairy cows under no heat stress with a temperature-humidity index (THI) below 68 (No-HS), mild HS (Mild-HS, 68 ≤ THI ≤ 79), or moderate HS (Mod-HS, 79 < THI ≤ 88). Error bars indicate SEM. ^**^P < 0.01

Figure 1 shows the yields of milk protein, and milk protein concentration of dairy cows under different HS. Under Mild-HS and Mod-HS, the yield and concentration of milk protein were all significantly lower (*P* < 0.001) than those in No-HS condition. Utilization and metabolism of total AA in the MG of dairy cows under varying HS are presented in Table 1. The mammary arterio-venous difference in total AA concentration was significantly higher (*P* = 0.003) under Mod-HS than that under Mild-HS. Supply (*P* = 0.045) and flow (*P* = 0.035) of total AA in MG were significantly higher under Mild-HS, but supply (*P* = 0.008) and flow (*P* = 0.014) of total AA in MG were significantly lower under Mod-HS compared to No-HS. Both total AA uptake (*P* = 0.004) and clearance rates (*P* = 0.019) were significantly lower in cows during Mod-HS than those under Mild-HS and No-HS. Total AA extraction rate was significantly higher (*P* = 0.001) under Mod-HS than under Mild-HS, whereas it tended to be lower (*P* = 0.067) under Mild-HS compared to No-HS. The ratio of milk protein yield to mammary AA supply in Mild-HS tended to be lower (*P* = 0.090) than that in No-HS and significantly lower (*P* = 0.001) than in Mod-HS, with no difference (*P* = 0.071) between No-HS and Mod-HS. No significant differences were found in the ratio of milk protein yield to mammary AA uptake among different conditions (*P* = 0.629).

**Table 1:** Amino acids (AA) uptake and utilization in the mammary gland of dairy cows under different heat stress

Item	Heat stress			SEM	*P* value
	No	Mild	Moderate		
Total AA concentration					
Artery (A), mg/L	301.29	287.41	294.15	11.781	0.545
Vein (V), mg/L	222.43	224.4	199.84	10.023	0.057
AV difference, mg/L	78.86^ab^	63.01^b^	94.31^a^	6.501	0.009
Milk, mg/L	324.61	332.47	327.28	7.483	0.602
Total AA supply and utilization					
Supply (A), kg/d	4.87^b^	6.54^a^	2.60^c^	0.555	< 0.001
Flow (V), kg/d	3.65^b^	5.21^a^	1.81^c^	0.490	< 0.001
Uptake, kg/d	1.23^a^	1.34^a^	0.79^b^	0.957	0.001
Output, kg/d	1.29^a^	1.17^b^	0.83^c^	0.028	< 0.001
U:O^1^	0.95	1.14	0.95	0.072	0.127
Clearance rate, L/h	239.32^a^	248.47^a^	169.11^b^	20.92	0.017
Extraction rate, %	26.54^ab^	21.96^b^	31.65^a^	1.873	0.005
Protein/Supply^2^, %	26.69^ab^	20.77^b^	32.23^a^	2.207	0.050
Protein/Uptake^3^, %	98.71	90.75	93.99	6.311	0.629

^1^U:O = AA uptake in MG (kg/d)/AA output in milk (kg/d)

^2^Ratio of protein yield to mammary supply of total AA

^3^Ratio of protein yield to mammary uptake of total AA

^a–c^Means within the same row with different superscripts are different (*P* < 0.05)

In general, the overall concentrations of free AA in milk were not different across treatments (Table S1), with only Cys showing a significant increase (*P* = 0.009) under Mod-HS compared with that under No-HS. Yield of AA in milk of dairy cows under varying heat stress are shown in Table S2. During Mild-HS and Mod-HS, the yield of AAs was significantly lower than those during No-HS (*P* < 0.01).

### Differentially abundant metabolites in milk under different HS

Following quality control, 1,749 metabolites were identified and quantified in milk. PCA showed clear separation between HS and No-HS groups, with PC1 explaining 12.4% (Mild-HS vs. No-HS) and 14.3% (Mod-HS vs. No-HS) of the variance (Fig. S1a–b), respectively. A total of 495 and 530 DAMs were detected in Mild-HS and Mod-HS relative to No-HS, respectively (Fig. S1g–h; Table S3).

**Fig. 2:**
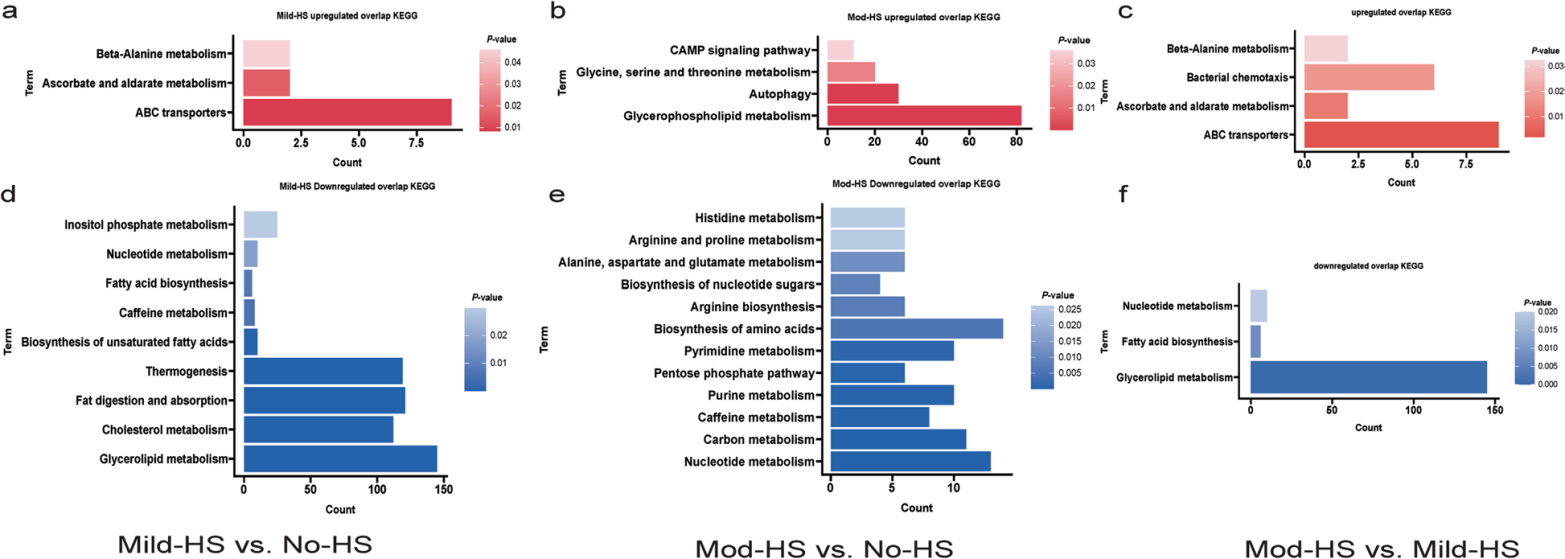
KEGG enrichment analysis of the upregulated and downregulated differentially abundant metabolites (DAMs) in milk of dairy cows under different heat stress (HS). **a–c** The significantly enriched KEGG pathways of upregulated DAMs in comparisons of Mild-HS vs. No-HS, Mod-HS vs. No-HS and Mod-HS vs. Mild-HS, respectively. **d–f** The significantly enriched KEGG pathways of downregulated DAMs in comparisons of Mild-HS vs. No-HS, Mod-HS vs. No-HS and Mod-HS vs. Mild-HS, respectively. The color gradient represents the *P* value, with darker shades indicating smaller *P* value. No-HS, no HS with a temperature-humidity index (THI) below 68; Mild-HS, mild HS (68 ≤ THI ≤ 79); Mod-HS, moderate HS (79 < THI ≤ 88)

Figure 2 presents the results of KEGG enrichment analysis of the upregulated and downregulated DAMs. In comparisons of Mild-HS vs. No-HS, the upregulated DAMs were mainly involved in ABC transporters, ascorbate and aldarate metabolism, and beta-alanine metabolism (Fig. 2a), while downregulated metabolites were enriched in pathways related to lipid metabolism (glycerolipid metabolism, cholesterol metabolism, fat digestion and absorption) and thermogenesis (Fig. 2d). In comparisons of Mod-HS vs. No-HS, the upregulated DAMs were significantly enriched in glycerophospholipid metabolism, autophagy, glycine, serine, and threonine metabolism, and the cAMP signaling pathway (Fig. 2b). Downregulated metabolites were mainly associated with nucleotide metabolism, carbon metabolism, purine metabolism, pentose phosphate pathway, pyrimidine metabolism, and biosynthesis of amino acids (Fig. 2e). The comparative metabolomics analysis between Mild-HS and Mod-HS groups revealed distinct metabolic responses to escalating HS (Table S3). In the Mod-HS vs. Mild-HS comparison, upregulated metabolites were significantly enriched in ABC transporters, ascorbate and aldarate metabolism, β-alanine metabolism (Fig. 2c). In contrast, downregulated metabolites in Mod-HS relative to Mild-HS were significantly enriched in pathways critical for glycerolipid metabolism (Fig. 2f).

### Milk proteomic under various HS

The detailed information in the DEPs in milk proteomic, including GO and KEGG pathway enrichments, is provided in Table S4. Heatmaps of the DEPs between No-HS and Mild-HS or Mod-HS are shown in Fig. S2a and b, respectively. In the comparison between the Mild-HS and No-HS (Fig. S2c), 8 proteins were found to be upregulated, while 81 proteins were downregulated. Similarly, in the comparison between the Mod-HS and No-HS (Fig. S2d), 23 proteins were upregulated, and 100 proteins were downregulated. However, no DEPs were identified between the Mild-HS and Mod-HS groups under the predefined stringent screening thresholds (log_2_FC > 1.5, FDR < 0.1, and* P* < 0.05).

**Fig. 3:**
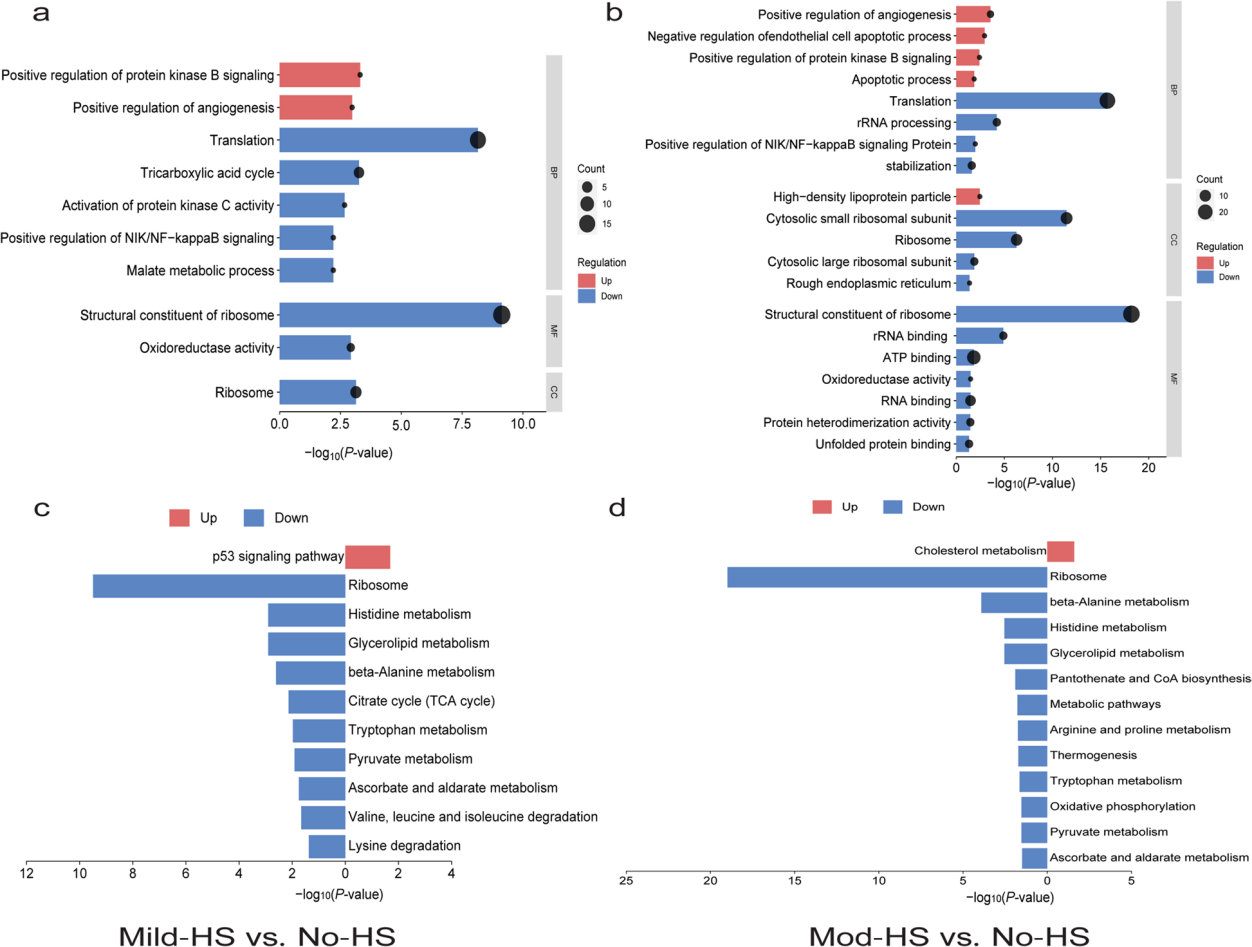
GO and KEGG enrichment analyses of up-regulated and down-regulated differentially expressed proteins (DEPs) in milk proteomics of dairy cows under different heat stress (HS). **a** GO enrichment analysis of upregulated DEPs between No-HS and Mild-HS groups. **b** GO enrichment analysis of downregulated DEPs between No-HS and Mod-HS groups. **c** KEGG pathway enrichment analysis of upregulated DEPs between No-HS and Mild-HS groups. **d** KEGG pathway enrichment analysis of downregulated DEPs between No-HS and Mod-HS groups. No-HS, no HS with a temperature-humidity index (THI) below 68; Mild-HS, mild HS (68 ≤ THI ≤ 79); Mod-HS, moderate HS (79 < THI ≤ 88)

Figure 3 presents the GO and KEGG enrichment analyses of upregulated and downregulated DEPs in milk proteomics. In the Mild-HS and No-HS comparison (Fig. 3a), the significantly upregulated proteins were associated with protein kinase B signaling and positive regulation of angiogenesis. In contrast, the significantly downregulated proteins were mainly enriched in pathways related to ribosomal structure and function, translation, ribosome, oxidoreductase activity, malate metabolic process, and positive regulation of NIK/NF-κB signaling (Fig. 3a). The significantly upregulated KEGG pathway was the p53 signaling pathway, whereas the downregulated pathways included ribosome, histidine metabolism, glycerolipid metabolism, beta-alanine metabolism, TCA cycle, tryptophan metabolism, pyruvate metabolism (ko00620), ascorbate and aldarate metabolism (Fig. 3c).

In the comparison between Mod-HS and No-HS, the significantly upregulated proteins were enriched in GO terms including angiogenesis, negative regulation of endothelial cell apoptotic process, and high-density lipoprotein particle. The downregulated GO pathways involved structural constituent of ribosome, translation, cytosolic small ribosomal subunit, ribosome, rRNA processing, ATP binding, protein stabilization, oxidoreductase activity, RNA binding, and unfolded protein binding (Fig. 3b). In the Mod-HS and No-HS comparison, the significantly upregulated KEGG pathway only included cholesterol metabolism, whereas the downregulated pathways were ribosome, histidine metabolism, glycerolipid metabolism, arginine and proline metabolism, thermogenesis, tryptophan metabolism, oxidative phosphorylation, pyruvate metabolism and ascorbate and aldarate metabolism (Fig. 3d).

### Integrated proteomic and metabolomic pathway analyses

**Fig. 4:**
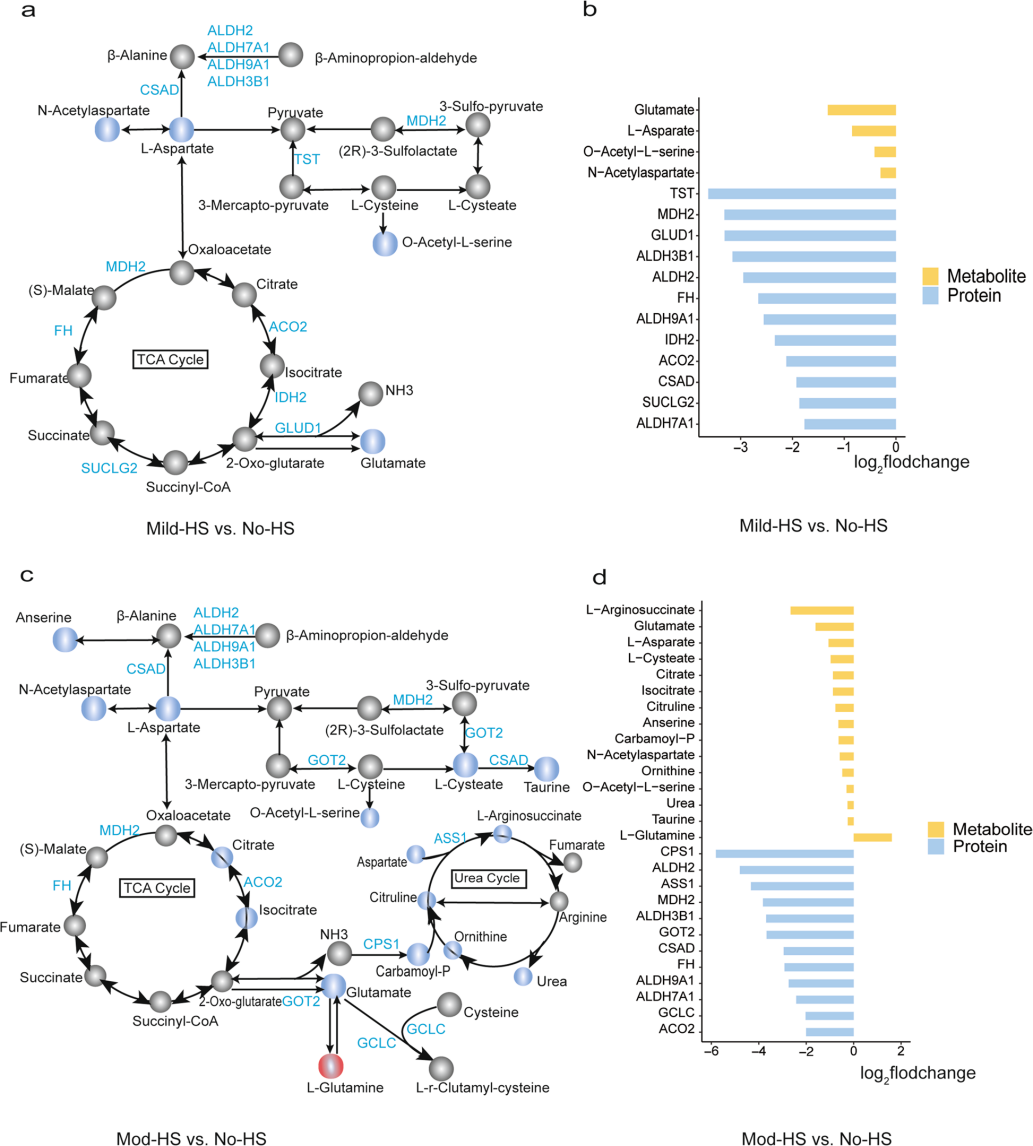
Illustration of the integrated proteomic and metabolomic KEGG pathway analyses of milk of dairy cows under different heat stress (HS). **a** and **c** Diagrams of pathway analyses of proteome and metabolome in Mild-HS vs. No-HS and in Mod-HS vs. No-HS, respectively. Circles represent metabolites. Blue and red colors indicate significantly downregulated and upregulated metabolites or proteins, respectively. Gray indicates non-significantly changed metabolites. The letters on the arrows represent the gene names for the identified proteins. Bar charts represent significantly altered metabolites and proteins in Mild-HS vs. No-HS (**b**) and Mod-HS vs. No-HS (**d**). No-HS, no heat stress (HS) with a temperature-humidity index (THI) below 68; Mild-HS, mild HS (68 ≤ THI ≤ 79); Mod-HS, moderate HS (79 < THI ≤ 88)

Integrated proteomic and metabolomic KEGG pathway analysis revealed coordinated metabolic alterations in AA metabolism and mitochondrial function under both Mild-HS and Mod-HS (Fig. 4). Under Mild-HS, several AA and their intermediates including glutamate and L-aspartate were downregulated. Correspondingly, multiple enzymes involved in AA metabolism and the TCA cycle (e.g., GLUD1, TST, ALDH family members, CSAD, MDH2, FH, IDH2, and ACO2) were also downregulated.

Figure 4c and d depict the integrated proteomic and metabolomic KEGG pathway analysis under Mod-HS, highlighting significant alterations in AA synthesis and related intermediates. Several metabolites including AA and their derivates involved in AA synthesis, TCA cycle (e.g., citrate and isocitrate), and the urea cycle (L-citrulline, L-arginosuccinate, carbamoyl phosphate, L-ornithine and urea). Under Mod-HS, enzymes involved in AA synthesis (ALDH2, ALDH3B1, ALDH9A1, CSAD, ALDH7A1, GCLC) were significantly downregulated. In the urea cycle, the key enzyme responsible for converting ammonia to carbamoyl phosphate, carbamoyl-phosphate synthase 1 (CPS1, ASS1). Mitochondrial enzymes related to the TCA cycle, including malate dehydrogenase (MDH2, FH, ACO2), were markedly decreased.

**Fig. 5:**
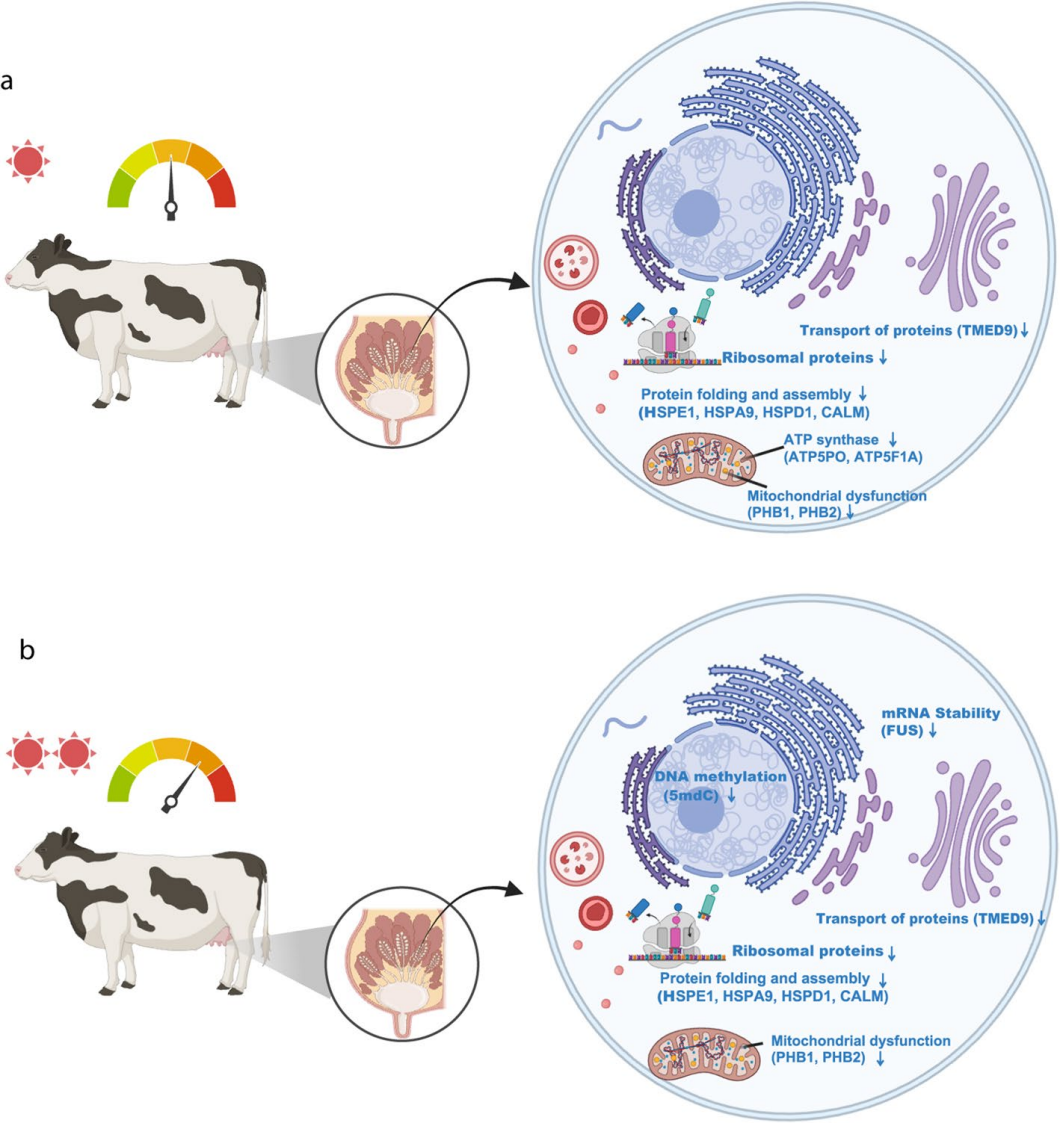
Diagrams illustrating the potential mechanisms of the effects of mild (**a**) and moderate (**b**) heat stress on milk synthesis in mammary epithelial cells. The biological processes related to protein trafficking, ribosomal proteins, protein folding and assembly, and mitochondrial dysfunction are shown inside the cell. The changes in DNA methylation and mRNA stability occur under moderate heat stress. Dashboard icons indicate differences in heat stress levels. The figures were created using BioRender

Figure 5 illustrates the potential underlying mechanisms through which Mild-HS or Mod-HS affects synthesis of milk composition in MECs. Under Mild-HS, protein trafficking pathway (TMED9) was inhibited, ribosomal protein synthesis was downregulated, and protein folding and assembly-related molecules (HSPs and CALM1) were downregulated. Additionally, early signs of mitochondrial dysfunction were evident, with ATP synthase components (ATP5PO, ATP5F1A) and mitochondrial markers (PHB1 and PHB2) showing mild alterations. In contrast, Mod-HS led to more severe disruptions, with further suppression of ribosomal protein synthesis, greater impairment of protein folding and assembly mechanisms, and aggravated mitochondrial dysfunction, resulting in significant disruptions to cellular energy metabolism. Furthermore, Mod-HS induced changes in DNA methylation (5mdC).

## Discussion

In the current study, we performed metabolomic and proteomic analyses of milk samples from dairy cows under different degrees of HS to reveal the HS effects on mammary protein synthesis at the levels of AA uptake and processes involved in protein synthesis. It is reported that reduced feed intake during HS contributed partially to the decline in milk yield [19]. Our study uniquely targeted the residual metabolic mechanisms independent of intake effects. The HS reduces milk protein and other milk component yields. Most of milk proteins are synthesized in MECs using the precursors (AA) derived from blood. Specific transporters take up AA to synthesize proteins in ribosomes on the rough endoplasmic reticulum, and proteins are accumulated in the Golgi for processing before secretion [20]. The main proteins synthesized in the MG are caseins, α-lactalbumin, and β-lactoglobulin, and some whey proteins in milk are directly derived from plasma. Milk protein synthesis is dependent on the arterial flow of AA to the MG, the extraction rate of AA by the MG through the AA transport system, and the metabolic and secretory activities of MECs [21]. Uptake of AA to MECs is one of the rate-limiting steps in milk protein synthesis, which depends on the concentration of AA in plasma and the rate of AA uptake by the MG. Although the effects of HS on milk protein synthesis have been widely reported [22, 23], fewer studies have systematically studied the effects of HS on the supply of precursors for milk protein synthesis and the specific processes of milk protein synthesis [22, 24].

It has been shown that HS reduces milk protein yield partly due to a deficiency of AA precursors in the MG of dairy cows, and that HS significantly reduced total AA concentrations in the blood compared to thermoneutral conditions [24, 25]. Although daily milk yield declined throughout late lactation, the instantaneous lactational metabolic demand remained for high milk yield (> 35 kg/d) during the Mild-HS period [4]. Our study showed no significant difference in total AA concentrations in arterial blood in HS conditions, but arterial total AA supply significantly increased during Mild-HS due to the increased MPF [4]. In our study, the total AA uptake did not show significant changes during Mild-HS, thus the decreased capacity of MECs to take up AA may be associated with suppressed expression of AA transporters. Consistently, the expression of the *SLC7A5*, a gene encoding a glutamine antiporter, was found to be reduced by HS in bovine MEC line MAC-T cells [26]. In contrast, we found that the supply and uptake of AA in MG were significantly decreased during Mod-HS, diminishing the availability of AA for milk protein synthesis. Additionally, HS can induce apoptosis and autophagy in MECs and reduce the number of MECs in the MG, which in turn decreases the cells available for milk protein synthesis [27]. In our previous study, an increase in the number of milk somatic cell counts was observed during Mod-HS [10], indicating a possible increase in MEC apoptosis. The number and viability of MECs are crucial for lactation persistency [28], thus an increase in MEC apoptosis can affect milk production in dairy cows.

Metabolomics integrates a wide range of metabolites, including AAs, FAs, organic acids, vitamins, and bioactive compounds [29]. Here, we integrated metabolomic and proteomic profiling to characterize metabolic alterations in response to HS in dairy cows. An integrated analysis of significantly altered metabolites and proteins during HS highlights changes in transcription, translation, and protein folding processes involved in protein synthesis. The ribosome, a large ribonucleoprotein complex, comprises two subunits that associate at the start of translation. The small subunit decodes the mRNA, while the large subunit catalyzes peptide bond formation. In mammalian cells, two groups of ribosomes are in the cytoplasm and on the endoplasmic reticulum, respectively. Ribosomal proteins account for half of all newly synthesized proteins in eukaryotic cells [30]. Global inhibition of protein synthesis is a hallmark of the cellular stress response. It is primarily attributed to the inhibition of translation initiation, with HS causing translational arrest in human and mouse cells [31]. The rate of protein synthesis depends on the proportion of mRNA molecules involved in translation, the number of ribosomes in the translationally active state, and the rate of elongation of these ribosomes [32]. Our proteomic results showed that some of ribosomal proteins, including 60S large subunits, 40S small subunits, and acidic ribosomal protein (RPLP0) were significantly decreased. This reduction of ribosomal proteins may directly affect the rate and efficiency of milk protein synthesis. Although SCC increased under HS as shown in our previous study [4], the abundance of ribosomal proteins in milk proteome was downregulated in both Mild-HS and Mod-HS conditions. This pattern likely reflects a suppression of ribosomal biogenesis and translational activity rather than a decrease in cellular protein release. Heat stress impairs protein synthesis in MECs by inhibiting the mTOR pathway and activating the unfolded protein response, leading to decreased ribosomal activity and protein translation [17]. Thus, despite the higher SCC, the overall translational machinery of mammary cells was repressed, resulting in reduced detection of ribosomal proteins in milk.

The heat shock response is a complex cellular program that induces significant changes in protein translation, folding, and degradation to mitigate the toxicity caused by protein misfolding [33]. In response to the HS, cells reduce overall protein synthesis but allow for the efficient translation of mRNA encoding heat shock proteins [34]. The 10-kDa heat shock protein (HSPE1 or HSP10) cooperates with 60-kDa heat shock protein (HSPD1) to ensure protein folding and maintain protein homeostasis [35]. Its abundance in milk was significantly reduced in response to Mild-HS, indicating that the function of helping newly synthesized proteins to fold and assemble correctly was decreased. Although classical HSP70 and HSP90 are typically upregulated during HS, the HSPs detected as downregulated in this study were other family members with distinct cellular functions. Their reduction may indicate feedback regulation or functional specialization under Mod-HS, rather than an overall suppression of heat shock response. In addition, HS appears to affect cell trafficking. Calmodulin is an essential component of the cellular second messenger system and plays a vital role in the Ca^2+^ signaling system. Spectrin alpha chain, non-erythrocytic 1 is involved in secretion by interacting with calmodulin to regulate the calcium-dependent movement of the cytoskeleton at the membrane [36]. Under Mild-HS, levels of non-erythrocytic 1 and calmodulin were significantly lower than those under No-HS, indicating that HS affected protein secretion. The transporter TMED9 located in the endoplasmic reticulum and Golgi network [37] was significantly lower in the Mild-HS group than under No-HS, indicating that HS also affected the transport of proteins between different organelles during protein synthesis.

Heat stress can cause mitochondrial fragmentation and decreases ATP levels, membrane potential, and antioxidant enzyme activity [38]. The gene *ATP5F1A* encodes a subunit of mitochondrial ATP synthase and the *ATP5PO* is associated with ATP synthesis [39]. In our study, these two ATP synthase subunits were significantly lower under Mild-HS than those under No-HS, indicating that mitochondrial function was impaired and ATP synthesis capacity was decreased, resulting in insufficient intracellular energy supply for protein synthesis. In addition, metabolic enzymes involved in the TCA cycle, such as fumarate hydratase (FH), malate dehydrogenase (MDH2), aconitase 2 (ACO2) and isocitrate dehydrogenase (IDH2) were significantly lower under Mild-HS than under No-HS, further limiting energy for protein synthesis.

Under Mod-HS conditions, cows could not adapt to the stress, and mammary AA uptake and supply decreased dramatically. More significant changes in metabolites and protein levels were observed in Mod-HS cows. The changes in TCA cycle, pentose phosphate pathway, and AA metabolism pathways were significantly enriched under Mod-HS. These significantly enriched pathways highlight a coordinated metabolic effort to enhance energy production, maintain redox homeostasis, and support protein synthesis and repair, all of which are vital for cellular adaptation and survival under thermal stress conditions. L-Glutamine was significantly increased under Mod-HS, indicating that glutamine may play a role in regulating stress. It was shown that L-glutamine specifically enhances HSP72 transcript abundance and HSF-1 DNA binding during heat shock [40] and can modulate the heat shock/stress response [41]. Consistently, milk glutamate concentration was also significantly decreased in cows under Mod-HS. Glutamate is an essential nitrogen donor in various transamination reactions and is required to synthesize other AA such as glutamine and proline. Its significant reduction indicates that the MECs may convert more glutamate to glutamine to support cytoprotective and stress responses.

In addition to decreasing the supply of precursors for milk protein synthesis, the processes of protein synthesis were also severely affected under Mod-HS. Proteome results showed that there was a significant reduction in ribosomal proteins (such as 40S ribosomal proteins RPS23, RPS8, RPS25, etc. and 60S ribosomal proteins RPL11, RPL28, RPL22, etc.) in cows under Mod-HS, similar to the condition under Mild-HS, which would directly affect ribosome assembly and mRNA translation efficiency. The significant reduction in heat shock proteins (HSPE1, HSPA9, and HSPD1) may lead to protein misfolding and dysfunction. Several metabolites involved in the urea cycle and arginine metabolism, including L-ornithine and L-arginine succinate, were significantly decreased, partially reflecting changes in nitrogen excretion and AA cycling pathways. This indicates that nitrogen metabolism and AA homeostasis are reprogrammed in response to stress or metabolic demands. Taken together, Mod-HS significantly affects nucleotide metabolism, energy metabolism, protein translation, folding, and transport, leading to a severe decrease in protein synthesis efficiency. The transition from Mild-HS to Mod-HS is marked by a shift from compensatory mechanisms (e.g., enhanced nutrient uptake via ABC transporters) to metabolic disruption, characterized by impaired mitochondrial function, reduced lipid synthesis. The downregulation of key metabolic pathways (e.g., fatty acid biosynthesis) and upregulation of ABC transporters highlight a trade-off between energy production and stress management. These findings suggest that the threshold for HS adaptation is exceeded in Mod-HS, leading to a cascade of metabolic and proteomic changes that negatively impact milk production and composition.

In this study, we used the metabolites and proteins in milk to represent the functions in MECs. The metabolites and proteins in milk are the final products of cellular activities of MECs, directly reflecting phenotypes as well as altered biological states and functions [42, 43]. While milk metabolites and proteins served as accessible proxies for assessing MEC function, their use is inherently indirect. Future studies should integrate milk omics data with direct MEC analyses, such as transcriptomics or proteomics of mammary tissues, to improve the robustness of these interpretations. In addition, our study utilized self-comparisons of cows at different HS conditions at different lactation stages. Although this design had advantage for reducing inter-individual variability, it may be confounded by stage-specific physiological changes. These alterations likely represent a metabolic adaptation aimed at reducing hepatic energy expenditure and maintaining internal homeostasis during heat stress. As our current study did not include pair-feeding treatments, it is acknowledged that the comparisons among the No-HS, Mild-HS, and Mod-HS groups may partially reflect differences in feed intake.

## Conclusions

In this study, we investigated the impact of varying degrees of HS on milk protein synthesis in dairy cows using metabolomics and proteomic analyses. Under Mild-HS, no significant changes occurred in AA uptake, although the supply of AA to the MG increased. However, the processes involving ribosomal proteins, heat shock proteins, protein transport and processing, and energy supply in the MG were affected, resulting in a decrease in milk protein yield beyond a reduce in feed intake. In contrast, the supply and uptake of AA decreased during Mod-HS, affecting the availability of AA precursors for milk protein synthesis in the MG. Additionally, Mod-HS significantly impacted several critical processes in the MG, including nucleotide metabolism, energy metabolism, protein translation, folding, and transport, leading to a severe decline in protein yield, without significantly altering overall protein/uptake efficiency. Overall, the changes in AA availability in mammary tissue and alterations in energy metabolism and protein synthesis-related enzymes during HS contribute to the reduction in milk protein synthesis. These findings from this study enhance our understanding of protein synthesis in the MG during HS, providing valuable insights into how HS reduces milk protein production in dairy cows.

## Supplementary Information

Additional file 1: Table S1. Concentrations of free amino acids in milk of dairy cows under varying heat stress. Table S2. Yield of free amino acids in milk of dairy cows under varying heat stress.

Additional file 2: Table S3. Differentially abundant metabolitesand KEGG Pathways.

Additional file 3: Table S4. Differentially expressed proteinsand GO and KEGG Pathways.

Additional file 4: Fig. S1. Quality control of metabolomic data and changes in differentially abundant metabolites (DAMs) expression. Fig. S2. Proteomic heatmap and volcano plot.

## Data Availability

All data generated or analyzed during this study are included in this published article.

## Abbreviations

AA: Amino acid
AV: Arterio-venous
DAMs: Differentially abundant metabolites
DEPs: Differentially expressed proteins
HIF-1α: Hypoxia-inducible factor 1α
HO-1: Heme oxygenase 1
HS: Heat stress
HSF: Heat shock transcription factor
HSP90: Heat shock protein 90
MECs: Mammary epithelial cells
MG: Mammary gland
Mild-HS: Mild heat stress
Mod-HS: Moderate heat stress
MPF: Mammary plasma flow
No-HS: No heat stress
