# Supplementary material for: Potential mechanism of the effect of heat stress on milk protein synthesis revealed by integrated metabolomic and proteomic analyses
Source: J Anim Sci Biotechnol. 2026 Feb 10;17:28. doi: 10.1186/s40104-025-01338-y (PMC12896087; doi:10.1186/s40104-025-01338-y)
Supplement: Supplementary file 1 — Additional file 1: Table S1. Concentrations of free amino acids in milk of dairy cows under varying heat stress. Table S2. Yield of free amino acids in milk of dairy cows under varying heat stress. [file 40104_2025_1338_MOESM1_ESM.docx]

Table S1. Concentrations of free amino acids in milk of dairy cows under varying heat stress

| Item1 | Heat stress | | | SEM | P value |
| --- | --- | --- | --- | --- | --- |
| (g/L) | No | Mild | Moderate |  |  |
| *EAA* | 15.094 | 15.444 | 15.232 | 0.330 | 0.594 |
| Arg | 1.096 | 1.133 | 1.144 | 0.027 | 0.254 |
| His | 0.856 | 0.880 | 0.864 | 0.021 | 0.531 |
| Ile | 1.574 | 1.609 | 1.579 | 0.033 | 0.492 |
| Leu | 3.230 | 3.289 | 3.254 | 0.073 | 0.726 |
| Lys | 2.631 | 2.686 | 2.610 | 0.059 | 0.441 |
| Met | 0.783 | 0.801 | 0.794 | 0.019 | 0.605 |
| Phe | 1.561 | 1.590 | 1.584 | 0.031 | 0.628 |
| Thr | 1.399 | 1.445 | 1.424 | 0.035 | 0.538 |
| Val | 1.964 | 2.011 | 1.979 | 0.044 | 0.266 |
| *NEAA* | 17.367 | 17.803 | 17.496 | 0.428 | 0.621 |
| Ala | 1.067 | 1.098 | 1.117 | 0.027 | 0.249 |
| Asp | 1.996 | 1.973 | 2.049 | 0.058 | 0.424 |
| Cys | 0.249^b^ | 0.261^ab^ | 0.278^a^ | 0.008 | 0.029 |
| Glu | 7.134 | 7.351 | 7.051 | 0.206 | 0.383 |
| Gly | 0.618 | 0.636 | 0.655 | 0.015 | 0.136 |
| Pro | 3.130 | 3.172 | 3.085 | 0.066 | 0.402 |
| Ser | 1.786 | 1.845 | 1.838 | 0.048 | 0.518 |
| Tyr | 1.387 | 1.467 | 1.423 | 0.044 | 0.266 |
| BCAA | 6.768 | 6.909 | 6.811 | 0.144 | 0.619 |

^1^EAA, Essential amino acids; NEAA, Non-essential amino acids; BCAA, Branched-chain amino acids (Valine, Isoleucine, and Leucine).

^a,b,c^ Means within the same row with different superscripts are different (*P* < 0.05).

Table S2. Yield of free amino acids in milk of dairy cows under varying heat stress

| Item^1^ | Heat stress | | | SEM | P value |
| --- | --- | --- | --- | --- | --- |
| (g/d) | No | Mild | Moderate |  |  |
| EAA | 598.80^a^ | 543.39^b^ | 387.61^c^ | 6.53 | <0.01 |
| Arg | 43.47^a^ | 39.86^b^ | 29.03^c^ | 0.45 | <0.01 |
| His | 33.97^a^ | 30.94^b^ | 21.99^c^ | 0.72 | <0.01 |
| Ile | 62.45^a^ | 56.61^b^ | 40.22^c^ | 1.28 | <0.01 |
| Leu | 128.13^a^ | 115.73^b^ | 82.80^c^ | 2.67 | <0.01 |
| Lys | 104.38^a^ | 94.49^b^ | 66.4^c^ | 2.17 | <0.01 |
| Met | 31.08ᵃ | 28.21ᵇ | 20.29ᶜ | 0.37 | <0.01 |
| Phe | 61.91ᵃ | 55.93ᵇ | 40.40ᶜ | 0.71 | <0.01 |
| Thr | 55.49ᵃ | 50.85ᵇ | 36.12ᶜ | 0.69 | <0.01 |
| Val | 77.92ᵃ | 70.78ᵇ | 50.35ᶜ | 0.78 | <0.01 |
| NEAA | 689.14ᵃ | 626.68ᵇ | 445.31ᶜ | 8.58 | <0.01 |
| Ala | 42.31ᵃ | 38.63ᵇ | 28.32ᶜ | 0.43 | <0.01 |
| Asp | 79.34ᵃ | 69.46ᵇ | 52.64ᶜ | 1.28 | <0.01 |
| Cys | 9.88ᵃ | 9.19ᵇ | 7.06ᶜ | 0.14 | <0.01 |
| Glu | 283.07ᵃ | 258.81ᵇ | 179.31ᶜ | 4.43 | <0.01 |
| Gly | 24.51ᵃ | 22.36ᵇ | 16.60ᶜ | 0.25 | <0.01 |
| Pro | 124.21ᵃ | 111.67ᵇ | 78.80ᶜ | 1.37 | <0.01 |
| Ser | 70.86ᵃ | 64.95ᵇ | 46.64ᶜ | 1.00 | <0.01 |
| Tyr | 54.96ᵃ | 51.61ᵇ | 35.94ᶜ | 0.90 | <0.01 |
| BCAA | 268.50a | 243.12b | 173.37c | 2.93 | <0.01 |

^1^ EAA, Essential amino acids; NEAA, Non-essential amino acids; BCAA, Branched-chain amino acids (Valine, Isoleucine, and Leucine).

^a,b,c^ Means within the same row with different superscripts are different (*P* < 0.05).
