# Supplementary material for: Potential mechanism of the effect of heat stress on milk protein synthesis revealed by integrated metabolomic and proteomic analyses
Source: J Anim Sci Biotechnol. 2026 Feb 10;17:28. doi: 10.1186/s40104-025-01338-y (PMC12896087; doi:10.1186/s40104-025-01338-y)
Supplement: Supplementary file 4 — Additional file 4: Fig. S1. Quality control of metabolomic data and changes in differentially abundant metabolites (DAMs) expression. Fig. S2. Proteomic heatmap and volcano plot. [file 40104_2025_1338_MOESM4_ESM.docx]

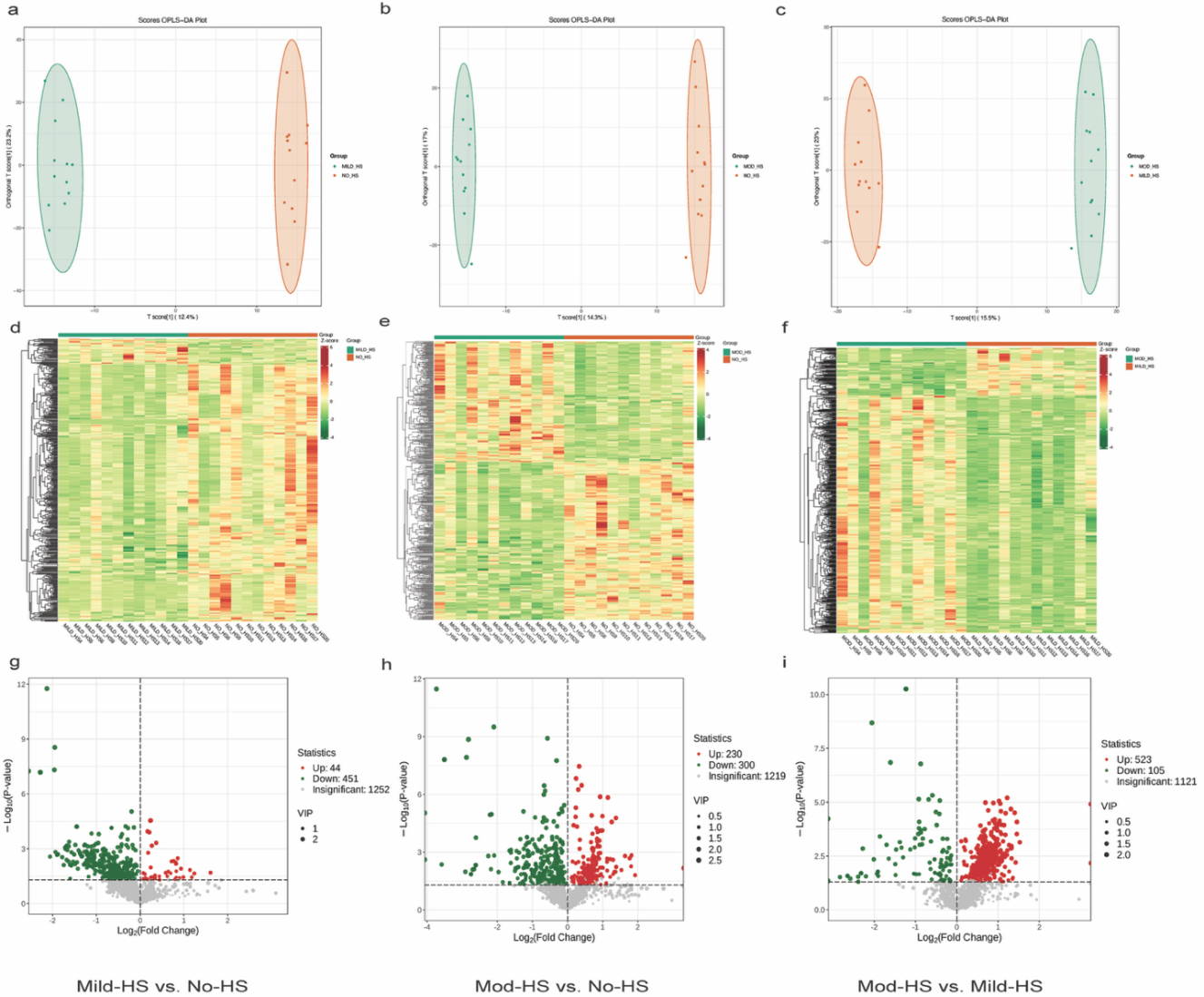


Figure S1. Quality control of metabolomic data and changes in differentially abundant metabolites (DAMs) expression. (a, b, c) OPLS-DA plots displaying the comparison of DAMs between Mild-HS vs. No-HS, Mod-HS vs. No-HS, and Mod-HS vs. Mild-HS respectively. (d, e, f) Heatmaps illustrating the comparison of DAMs across different groups. (g, h, i) Volcano plot of DAMs between cows under different heat stress (HS). Red color indicates upregulated metabolites, and green indicates downregulated metabolites.


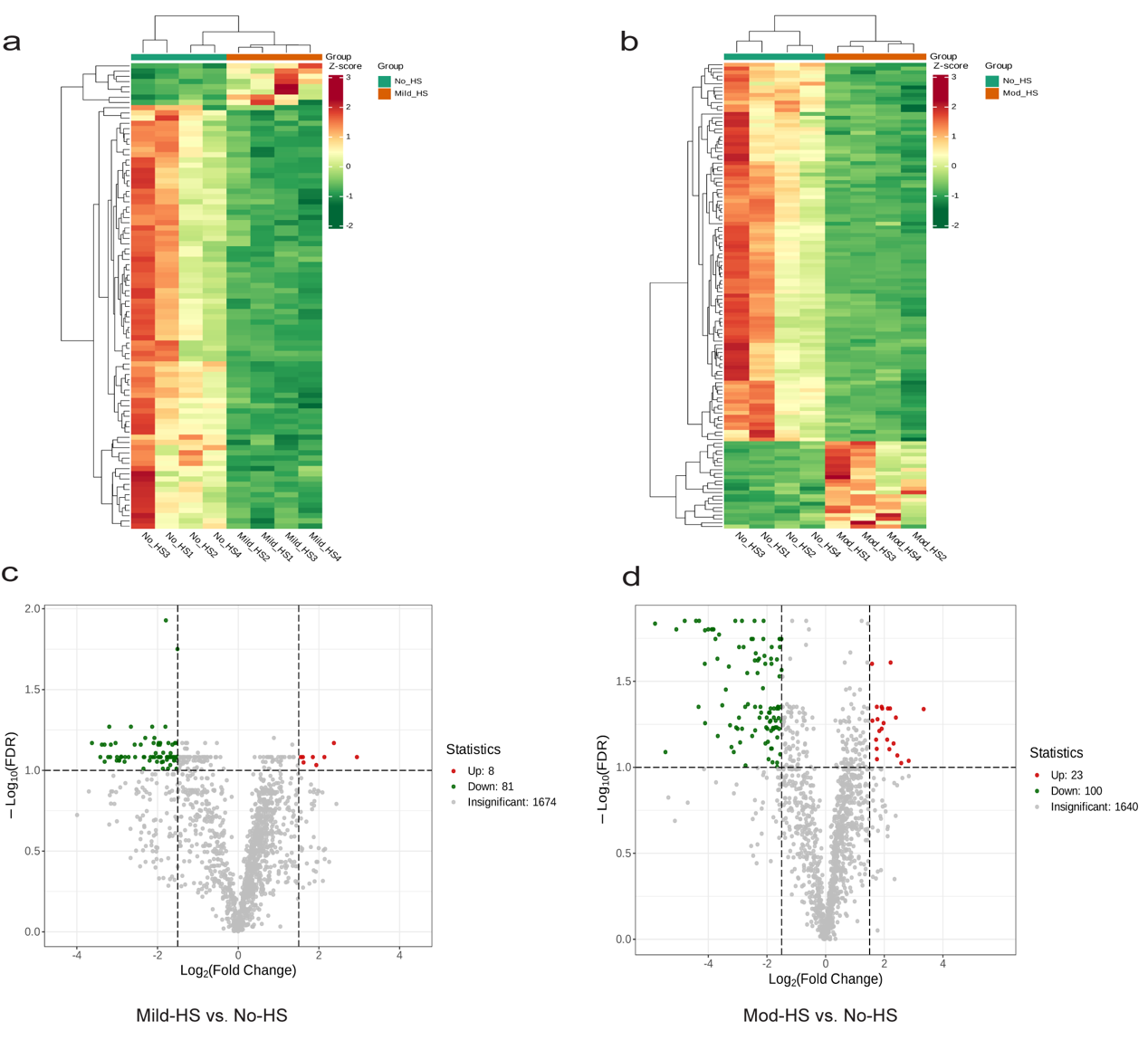


Figure S2. Proteomic heatmap and volcano plot. (a) Heatmap of differentially expressed proteins (DEPs) between the No-HS and Mild-HS groups; (b) Heatmap of DEPs between the No-HS and Mod-HS groups; (c) Volcano plot of upregulated and downregulated proteins between the No-HS and Mild-HS groups; (d) Volcano plot of upregulated and downregulated proteins between the No-HS and Mod-HS groups. No-HS, no HS with a temperature-humidity index (THI) below 68; Mild-HS, mild HS (68 ≤ THI ≤ 79); Mod-HS, moderate HS (79 < THI ≤ 88).
